# Dilated and Noncompaction Cardiomyopathy in a Pediatric Patient With Maternal *CASZ1* Variant

**DOI:** 10.1016/j.jaccas.2026.108389

**Published:** 2026-06-01

**Authors:** Lauren Sekiguchi, Michael Nguyen-Truong, Kristy Y. Lin, David P. Chan

**Affiliations:** a Carle Illinois College of Medicine, Urbana, Illinois, USA; b Carle Foundation Hospital, Urbana, Illinois, USA

**Keywords:** cardiomyopathy, genetic disorders, genetics, left ventricle, murmur, tachycardia

## Abstract

**Background:**

The *CASZ1* gene expresses a cardiomyocyte transcription factor implicated in early heart morphogenesis. *CASZ1* variants have been associated with the development of dilated cardiomyopathy (DCM) and noncompaction cardiomyopathy (NCCM), although reported cases are limited.

**Case Summary:**

A 3-month-old boy with no family history of cardiomyopathy presented with a murmur and persistent tachycardia. Echocardiogram showed moderate global left ventricular hypokinesis with trabeculations and mild left atrial dilation. Genetic testing identified an autosomal dominant *CASZ1* variant in exon 15, c.3088del:p.(His1030Thrfs∗120), inherited from his asymptomatic mother. Although he is clinically doing well at age 18 months, he will need lifelong management.

**Discussion:**

Our case highlights the discovery of a novel *CASZ1* variant leading to DCM and NCCM, emphasizing the importance of genetic testing in patients with no known family history.

**Take-Home Message:**

Genetic testing is a critical component of the workup of unexplained DCM and NCCM in pediatric patients.

## Visual Summary

**Figure:**
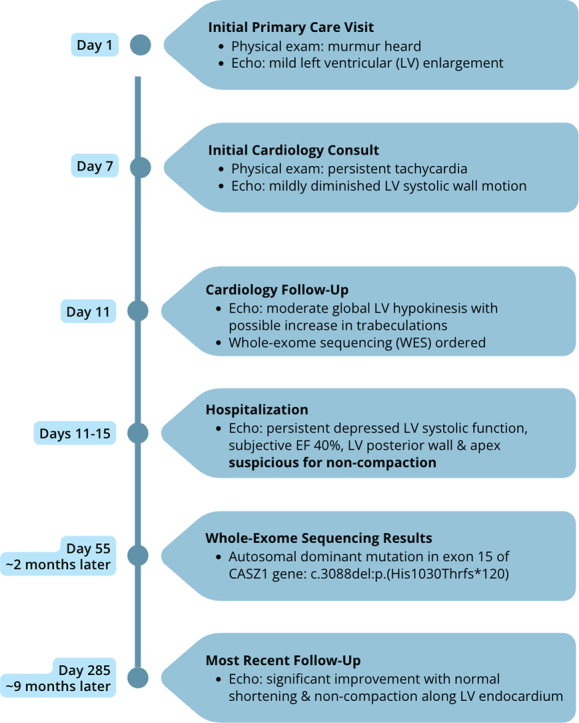

Dilated cardiomyopathy (DCM) represents approximately 50% of pediatric cardiomyopathies and is characterized by ventricular dilation with reduced systolic function.^1^ DCM can be complicated by noncompaction cardiomyopathy (NCCM), a rare condition often affecting the left ventricle, which results in the formation of an inner noncompacted layer with prominent trabeculations.^2^ Clinical manifestations of DCM and NCCM vary; some patients are asymptomatic, whereas others experience dyspnea, feeding difficulties, or even sudden cardiac death.^3^ Patients with both DCM and NCCM tend to have poorer prognosis, including increased risk of heart failure.^3^ Therefore, proper diagnosis is crucial to optimize patient outcomes.

Genetic testing plays a significant role in the diagnosis of pediatric cardiomyopathies. Familial cardiomyopathy has been found in 30% to 50% of DCM cases and 43% of noncompaction cases.^4^^,^^5^ Castor zinc finger 1 (*CASZ1*), a transcription factor, plays an important role in early cardiac development.^6^ In humans, *CASZ1* variants have been associated with DCM and NCCM, but reported cases are limited. We discuss the case of a 3-month-old boy diagnosed with concomitant DCM and NCCM attributed to an inherited *CASZ1* gene variant.

## History of presentation, past medical history, and differential diagnosis

**Figure 1:**
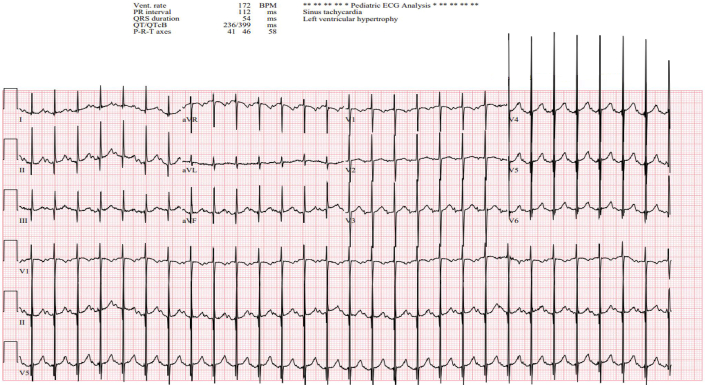
Initial Electrocardiogram Electrocardiogram obtained during initial cardiology consult revealed sinus tachycardia.

**Figure 2:**
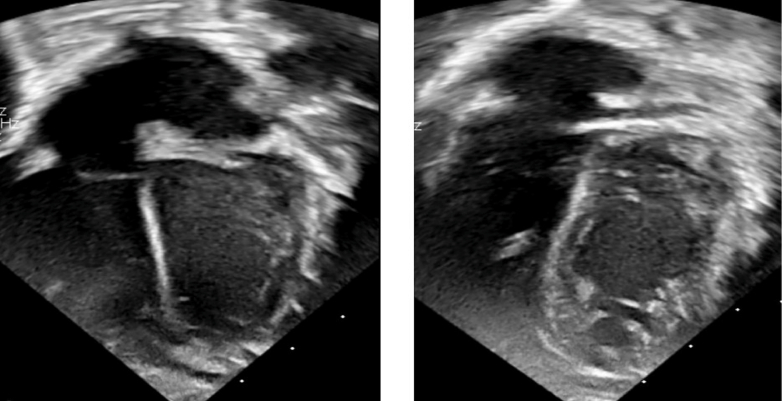
Control vs Noncompaction Echocardiogram Echocardiogram (apical 4-chamber view) depicting normal cardiac anatomy in age-matched control (left) vs noncompaction cardiomyopathy in patient (right).

The patient was a 3-month-old boy with no known family history of cardiomyopathy who presented with a murmur. Cardiology was consulted. There were no reported feeding difficulties, respiratory distress, cyanosis, excessive diaphoresis, failure to thrive, or recent infection. Electrocardiogram showed persistent tachycardia with heart rate (HR) of 172 beats/min. Echocardiogram revealed mildly diminished left ventricular (LV) systolic wall motion and mild LV enlargement. There was no evidence of a patent ductus arteriosus (Figure 1, Table 1). Twenty-four-hour Holter monitor revealed sinus tachycardia with a mean HR of 164 beats/min and maximum HR of 221 beats/min. At follow-up 4 days later, the patient's parents reported increased respiratory rate but denied any feeding difficulties, syncope, or excessive diaphoresis. Physical examination demonstrated persistent tachycardia with HR of 172 beats/min. Repeat echocardiogram showed moderate global LV hypokinesis with trabeculations and mild left atrial dilation without mitral regurgitation (Figure 2, Table 1). Differential diagnoses included structural congenital anomalies such as anomalous left coronary artery from the pulmonary artery, tachycardia-induced cardiomyopathy, genetic cardiomyopathy, metabolic disorder, postviral myocarditis, Barth syndrome, or idiopathic changes. Whole exome sequencing was ordered to investigate the genetic basis of cardiomyopathy. Given the complexity of the workup and need to start medication, the patient was admitted to the hospital.

## Investigations and management

**Figure 3:**
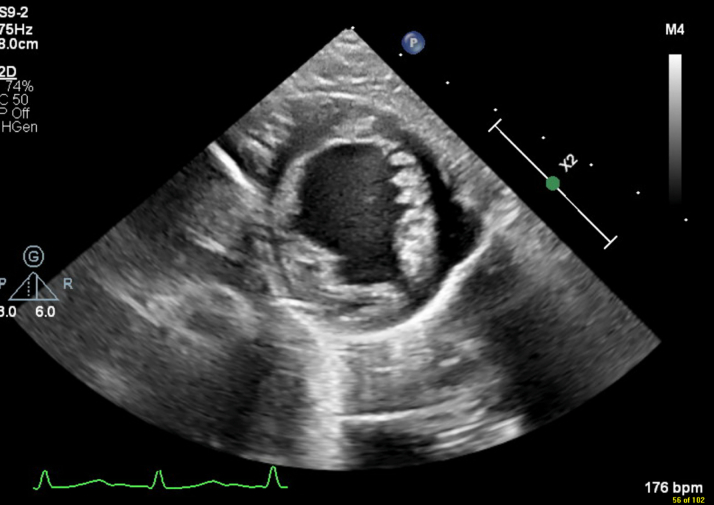
Initial Echocardiogram Echocardiogram (parasternal short axis view) from initial visit.

**Figure 4:**
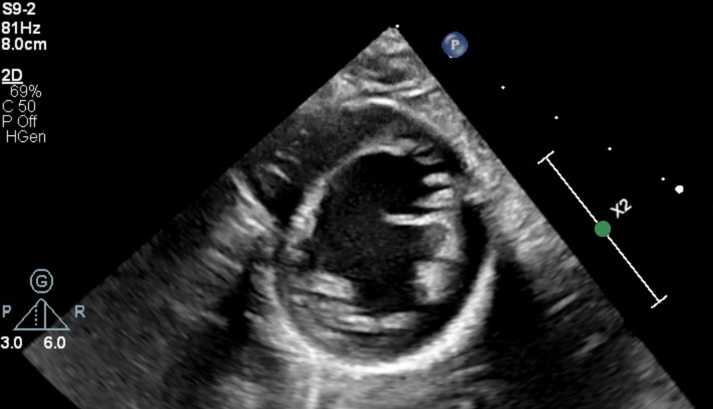
Hospital Follow-Up Echocardiogram Echocardiogram (parasternal short axis view) from first follow-up visit after hospitalization.

On admission, the patient presented with tachycardia, diaphoresis on his back, and fussiness when not in an upright position. He was not in acute distress. There was no observable pallor, cyanosis, murmurs, or hepatomegaly. Blood pressure was 106/75, HR was 182 beats/min, rectal temperature was 36.9 °C, respiratory rate was 39 breaths per minute, and oxygen saturation was 100%. Weight was 6.54 kg (30th percentile) and height was 62 cm (22nd percentile). B-type natriuretic peptide was 292 pg/mL (reference range <100 pg/mL). Chest x-ray showed projected prominence of the cardiothymic silhouette. Repeat electrocardiogram confirmed narrow QRS tachycardia. Repeat echocardiogram demonstrated DCM, moderately depressed LV systolic function, and mild dilation with an ejection fraction of 35% to 40% (Figure 3, Table 1). Given these findings, carvedilol 0.1 mg/kg twice daily and enalapril 0.1 mg/kg twice daily were started. Workup included electrocardiogram, echocardiogram, computed tomography angiography (CTA), myocarditis/pericarditis panel, parvovirus/adenovirus/enterovirus panel, respiratory pathogen panel, carnitine panel, and cardiomyopathy gene panel. Myocarditis/pericarditis, parvovirus/adenovirus, and carnitine panels were unremarkable. Respiratory pathogen panel was positive for rhinovirus/enterovirus, but he was asymptomatic. The next day, carvedilol was increased to 0.2 mg/kg twice daily, enalapril was increased to 0.2 mg/kg twice daily, and digoxin 10 μg/kg twice daily was started. This was because of heart failure symptoms and sinus tachycardia overnight, with HR of 182 beats/min. Milrinone 0.25 μg/kg/min was started before the CTA, which demonstrated high takeoff of the left main coronary artery and ruled out anomalous left coronary artery from the pulmonary artery. As telemetry over admission showed variability and persistent tachycardia with an HR of 163 beats/min, carvedilol was increased to 0.3 mg/kg twice daily, enalapril was increased to 0.3 mg/kg twice daily, and digoxin was continued at 10 μg/kg twice daily. The next day, tachycardia resolved with an HR of 152 beats/min, but repeat echocardiogram revealed persistently depressed LV systolic function with subjective ejection fraction of 40% and LV posterior wall and apex suspicious for noncompaction but no congenital anomalies (Figure 4, Table 1). As a result, carvedilol was increased to 0.4 mg/kg twice daily. Furosemide 1 mg/kg twice daily and spironolactone 0.5 mg/kg/d were started. Enalapril 0.3 mg/kg twice daily and digoxin 10 μg/kg twice daily were continued. He was discharged the next day on carvedilol 0.4 mg/kg twice daily, enalapril maleate 0.3 mg/kg twice daily, digoxin 10 μg/kg twice, and furosemide 1 mg/kg twice daily with improved symptoms.

## Outcome and follow-up

**Figure 5:**
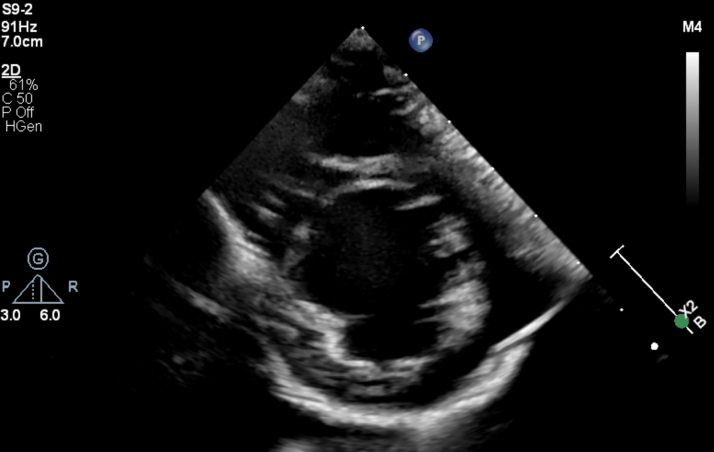
Most Recent Echocardiogram Echocardiogram (parasternal short axis view) from most recent follow-up, approximately 15 months after hospitalization.

At his follow-up with cardiology 4 days after discharge, he remained stable, but echocardiogram showed a moderate degree of LV dysfunction (Figure 5, Table 1). Two weeks later, comprehensive cardiomyopathy gene panel revealed 3 variants of unknown significance: LMNA c.524C>T (p.Ala175Val), TTN c.95484T>G (p.Cys31828Trp), and PCCA c.1519G>A (p.Val507Met). One month after discharge, whole exome sequencing identified a rare, likely pathogenic autosomal dominant *CASZ1* variant in exon 15: c.3088del:p.(His1030Thrfs∗120). This variant was traced back to the patient's mother, who carries the same genetic test result. Echocardiogram on the patient's mother is pending. As of his last evaluation 15 months after initial presentation, the patient is clinically doing well but will need lifelong management. He is currently taking furosemide 10 mg daily, enalapril 1 mg twice daily, and carvedilol 3 mg twice daily, and was weaned off digoxin because of improvement in his symptoms. His most recent echocardiogram with ongoing treatment showed minimal LV dysfunction and noncompaction along the LV endocardium, with an LV internal dimension at end-systole of 2.3 cm (*z*-score 2.41), LV internal dimension at end-diastole of 3.3 cm (*z*-score 1.65), and fractional shortening of 30.8% (*z*-score −2.2).

## Discussion

When investigating the etiology of unexplained DCM and NCCM in a pediatric patient, it is important to maintain a broad differential. Differential diagnoses included structural congenital anomalies such as anomalous left coronary artery from the pulmonary artery, tachycardia-induced cardiomyopathy, genetic cardiomyopathy, metabolic disorder, postviral myocarditis, Barth syndrome, or idiopathic changes. These considerations warranted a comprehensive workup, including electrocardiogram, echocardiogram, B-type natriuretic peptide measurement, CTA, myocarditis/pericarditis panel, parvovirus/adenovirus/enterovirus panel, respiratory pathogen panel, carnitine panel, cardiomyopathy gene panel, and whole exome sequencing. This workup is in line with the American Heart Association's current guidelines, which encourage a systematic approach.^7^ These recommendations include diagnosis via echocardiogram with further characterization via cardiac magnetic resonance imaging, comprehensive genetic evaluation, and exclusion of secondary causes such as congenital heart disease.^7^ Although cardiac magnetic resonance imaging serves as the gold standard for NCCM, this test was deferred due to several considerations, including the patient's lack of symptoms, low clinical utility, and need for general anesthesia.

In pediatric patients with DCM, the American Heart Association recommends angiotensin-converting enzyme inhibitors or angiotensin II receptor blockers, beta-blockers, mineralocorticoid receptor antagonists, and diuretics for volume overload.^7^ By contrast, the management of NCCM is empirical, with improvement of LV function reported with the use of beta-blockers, angiotensin-converting enzyme inhibitors, and/or angiotensin II receptor blockers.^8^ In our case, use of enalapril, carvedilol, spironolactone, furosemide, and digoxin resulted in improvement in ventricular function and shortening.

Although guidelines exist for the diagnosis and management of DCM and NCCM, respectively, there are very few documented cases describing the evaluation and outcomes of patients with concomitant DCM and NCCM attributed to *CASZ1* variants. Furthermore, none of these cases specifically validate the guidelines in this context. To our knowledge, there are only 2 patients who have presented with both DCM and NCCM attributed to *CASZ1* variants. Al-Hassnan et al^9^ reported a homozygous p.Ser237Cys variant in a girl with consanguineous parents at the age of 5 months. She was alive at 6.5 years and had not required a heart transplant at that time.^9^ Guo et al^10^ identified a de novo heterozygous frameshift variant, pVal815Profs∗14, in a boy admitted for fever and cough at the age of 11 months. X-ray revealed marked cardiomegaly, and he died from ventricular fibrillation on hospital day 2.^10^ In comparing the outcomes of our patient with that of Guo et al,^10^ who also had a frameshift *CASZ1* variant, it appears that the location may also significantly influence the degree of cardiac dysfunction and prognosis. Our patient, who also presents with both DCM and NCCM, carries a novel *CASZ1* variant that critically expands on the clinical presentation, diagnosis, and management of this condition. Furthermore, the patient's presentation in the setting of asymptomatic findings in the mother adds to the unique features of this case and calls for further investigation. By providing a firsthand account in support of the current guidelines, our case emphasizes the utility of genetic evaluations in identifying the origin of dysfunction, which ultimately revealed implications extending beyond the patient himself.

Although further investigation is required to fully elucidate the pathogenicity of the *CASZ1* variants described thus far, the collective outcomes of these cases implicate *CASZ1* as an important factor in the development of DCM and NCCM. In this particular case, we hypothesize that the patient's frameshift variant resulted in premature truncation of the *CASZ1* protein and subsequent loss of protein function, leading to abnormal cardiac development and dilated cardiomyopathy.^10^ In murine models, *CASZ1* knockout resulted in cardiac noncompaction and ventricular septal defect, suggesting the importance of this gene in cardiac morphogenesis and contraction.^11^ Through genetic mapping of murine cardiomyocytes, Dorr et al^12^ demonstrated a potential underlying mechanism that loss of *CASZ1* led to prolonged or arrested G1 phase with reduction in DNA synthesis and cardiac mitotic index. Taken together, a prolonged G1 phase would result in impaired DNA synthesis (S phase), defective cardiomyocyte maturation, and ultimately abnormal cardiac tissue development. For our patient, these findings may implicate this particular frameshift *CASZ1* variant in aberrant cardiac morphogenesis and subsequent development of DCM and NCCM.

The following considerations will help bolster our understanding of the underlying mechanism and clinical relevance of this variant. Future studies to elucidate transcriptional activity, such as in vitro analyses, will be valuable to our understanding of this specific *CASZ1* variant on cardiac development and clinical course from birth through adulthood. However, it may be difficult to fully extrapolate this patient's genetic findings to all patients because of the variable expressivity associated with this gene. Furthermore, functional analyses on this novel variant would help to inform and enhance the long-term predictability in the management of this patient.

**Visual Summary:**
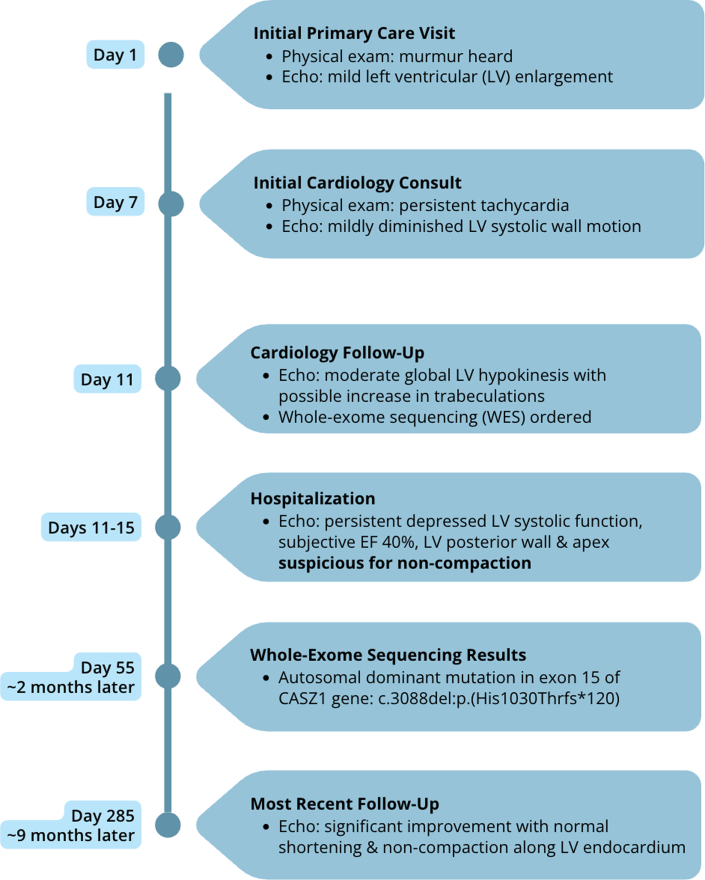
Clinical Course

## Conclusions

*CASZ1* plays an important role in the development of DCM and NCCM; however, as evidenced by the variable clinical presentation of our patient and his mother, much remains unknown about the penetrance of autosomal dominant *CASZ1* variants. Although some patients present with systolic dysfunction and LV shortening, others may be asymptomatic. Acknowledgment of the inheritance of *CASZ1* variants and diversity of presentation is crucial when discussing the implications of these results with families, as this information may play a critical role in screening of current relatives and family planning for future children.

**Take-Home Messages**

• This case highlights the diversity of clinical presentations of *CASZ1* variants, which may result in the development of both DCM and NCCM in a single patient.
• Genetic testing is a critical component of the workup of unexplained DCM and NCCM in pediatric patients, as the results can have important implications for both the children and their families.
• Frameshift variants may result in premature truncation of the *CASZ1* protein and subsequent loss of protein function, leading to abnormal cardiac development and DCM.

**Table 1:** Key Echocardiogram Measurements

Echocardiogram	Key Measurements
#1 (initial visit)	1. LV end-diastolic dimension (LVDd): 3.2 cm (*z*-score 3.65) 2. LV end-systolic dimension (LVDs): 2.4 cm (*z*-score 5.42) 3. Stroke volume: 18.2 mL 4. Fractional shortening (FS): 25.4% (*z*-score −5.04)
#2 (at follow-up 4 days later)	1. LVDd: 2.4 cm (*z*-score 0.03) 2. LVDs: 2 cm (*z*-score 2.67) 3. FS: 20.1% (*z*-score −7.84)
#3 (on admission)	1. LV end-diastolic volume (LVEDV): 16.1 mL 2. LV end-systolic volume (LVESV): 10.3 mL 3. Calculated ejection fraction (EF): 36%
#4 (mid-hospitalization)	1. LVEDV: 9.5 mL 2. LVESV: 5.1 mL 3. Calculated EF: 46.3%
#5 (first follow-up after discharge)	1. LVDd: 2.6 cm (*z*-score 0.5) 2. LVDs: 2 cm (*z*-score 2.72) 3. FS: 22.9% (*z*-score −6.26)
#6 (most recent)	1. LV internal dimension at end-systole: 2.3 cm (*z*-score 2.41) 2. LV internal dimension at end-diastole: 3.3 cm (*z*-score 1.65) 3. FS: 30.8% (*z*-score −2.2)

## Funding Support and Author Disclosures

The authors have reported that they have no relationships relevant to the contents of this paper to disclose.
